# Hepatoid Adenocarcinoma of the Stomach: Current Perspectives and New Developments

**DOI:** 10.3389/fonc.2021.633916

**Published:** 2021-04-12

**Authors:** Ruolan Xia, Yuwen Zhou, Yuqing Wang, Jiaming Yuan, Xuelei Ma

**Affiliations:** ^1^ Department of Biotherapy, Cancer Center, West China Hospital, Sichuan University, Chengdu, China; ^2^ West China School of Medicine, Sichuan University, Chengdu, China; ^3^ The Center of Gerontology and Geriatrics, West China Hospital, Sichuan University, Chengdu, China

**Keywords:** hepatoid gastric carcinoma, pathology, diagnosis, prognosis, treatment

## Abstract

Hepatoid adenocarcinoma of the stomach (HAS) is a rare malignant tumor, accounting for only 0.17–15% of gastric cancers. Patients are often diagnosed at an advanced disease stage, and their symptoms are similar to conventional gastric cancer (CGC) without specific clinical manifestation. Morphologically, HAC has identical morphology and immunophenotype compared to hepatocellular carcinoma (HCC). This is considered to be an underestimation in diagnosis due to its rare incidence, and no consensus is reached regarding therapy. HAS generally presents with more aggressive behavior and worse prognosis than CGC. The present review summarizes the current literature and relevant knowledge to elaborate on the epidemic, potential mechanisms, clinical manifestations, diagnosis, management, and prognosis to help clinicians accurately diagnose and treat this malignant tumor.

## Introduction

Hepatoid adenocarcinoma of the stomach (HAS), the Primer's focus, is a scarce primary extrahepatic malignant neoplasm. The estimated annual incidence of HAS is 0.58–0.83 cases per million individuals. Most tumors have metastasized at diagnosis with a poor prognosis due to their aggressive behavior (1, 2). Hepatoid adenocarcinoma(HAC) has been reported to occur in the stomach (3), esophagus (4, 5), duodenum (6), jejunum (2), colon (7), peritoneum (8), pancreas (9–13), lung (14), ovary (15, 16), gallbladder (17), uterus (16, 18) and other sites (19). Of these locations, the stomach is the most common site of HAC. Histologically, HAC has similar morphology and immunohistochemistry to hepatocellular carcinoma (HCC). This is considered to be an underestimation in diagnosis due to its rare incidence, and no consensus is reached regarding therapy (20). Although numerous cases and a small sample of retrospective reports on HAS have been reported over the years, it has not been sufficiently identified. Herein, to deepen the comprehensive understanding of HAS, we elaborate on the epidemic, potential mechanisms, clinical manifestations, diagnosis, management, and prognosis of this neoplasm based on current literature and relevant materials to assist clinicians in diagnosing and treating this disease.

## Epidemiology

HAS is a rare neoplasm and the annual incidence of HAS is approximately 0.58–0.83 cases per million people (2, 21). It is also a scare entity with an inconstant reported incidence between 0.17% and 15.0% in all gastric carcinomas across several studies (20, 22). A large number of HAS case reports come from the Asian region, mainly from the Japanese and Chinese cohort (22). According to previously published reports, HAS predominantly occurred in around 65 years old male patients (21, 23). Although no specific risk factors have been reported to influence the occurrence and progression of HAS positively, several cases described patients diagnosed as HAS with HBsAg seropositivity (8, 24).

## Pathogenesis

The exact molecular mechanism of HAS remains unclear. A possible hypothesis is that based on the stomach and liver, with a common embryonic and histological origin, originating from the endoderm and the primitive foregut during the development of the embryo (25–27). The major genotypes of gastric malignancy have been defined by The Cancer Genome Atlas (TCGA) Research Network as Epstein–Barr virus-positive (EBV), microsatellite-instable (MSI), genomically stable tumors (GS), and chromosomally instability tumors (CIN): HAS is excluded from any of these due to its scarcity and characteristics of geographical distribution (28). Nevertheless, HASs are genetically heterogeneous groups with a majority of HAC are “CIN” and a small number of HAC with “MSI” (29, 30). It has been speculated that HAS is the result of trans-differentiation, transitioning from the intestinal type to hepatoid phenotypic (31); and the emergence of Alpha-fetoprotein (AFP) leading to hepatoid focus in gastric adenocarcinoma, may as a result of dedifferentiation of cancer cells into HAC progenitor cells. The HAS, obtaining AFP phenotype expression, may evolve into various microscopic histological morphology, including enteroblastic carcinoma and poorly differentiated medullary carcinoma through genetic divergence and evolution (32). Furthermore, HAS appears as invasive cancer with high deletion of alleles and extensive loss of heterozygosity (LOH), where some tumor suppressor genes are located in Ref. (32).

## Diagnosis

### Pathology

Pathology is the “gold standard” for diagnosing the HAS. Macroscopically, according to Borrmann’s classification, majority of patients were type III with poor differentiation and elevated serum AFP levels. The most common primary locations of these tumors were the antrum and body (26, 33). Microscopically, HAS was defined as a tumor with the resemble features of hepatoid adenocarcinomas with hematoxylin and eosin (H&E) stains, consisting of large eosinophilic cells with a similar morphology to HCC, which exhibiting trabecular or solid nested arrangement, separated by sinusoidal vascular channels (33–35). Assorted degrees differentiation of clear cells imitating embryonic foregut epithelium can also be found, indicating the differentiation of fetal enteroblastic. Nevertheless, precise diagnosis of HAC was difficult based on findings in histology statistics alone, with a low positive rate of 9.3% (36). Further assistance like immunohistochemistry (IHC) stains was regularly performed for diagnosis (37).

### Immunohistochemistry

**Figure 1:**
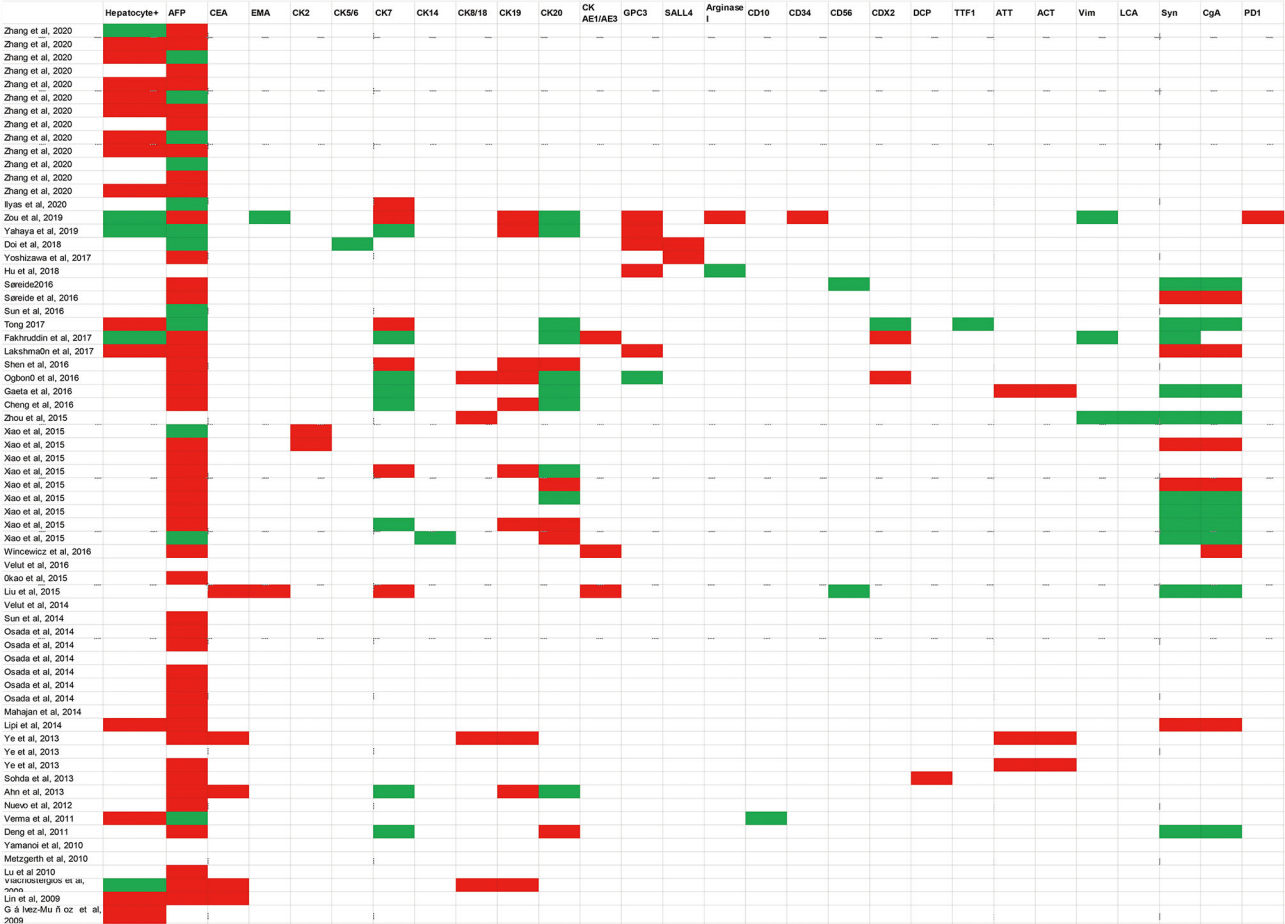
Summarized a variety of immunohistochemistry markers in published case reports. Diagnostic markers include Hepatocyte+, AFP, CEA, EMA, CK2, CK5/6, CK7, CK14, CK8/18, CK19, CK20, CK AE1/AE3, GPC3, SALL4, Arginase I, CD10, CD34, CD56, CDX2, DCP, TTF1, ATT, ACT, Vim, LCA, Syn, CgA, PD1.White blocks mean this examination has not been mentioned in case reports; green blocks represent negative results; red blocks represent positive results. AFP Alpha-fetoprotein; CEA Carcinoembryonic antigen; EMA, Epithelial cell membrane antigen; CK, Cytokeratin 2; GPC3, Glypican 3; SALL4, Sal-like protein 4; DCP, Des-gamma-carboxyprothrombin; TTF1, Thyroid transcription factor-1; ATT, A-1-antitrypsin; ACT, A-1-antichymotrypsin; Vim: Vimentin; LCA, Leucocyte common antigen; Syn Synaptophysin; CgA, Chromogranin A; PD-1 Programmed cell death protein 1.

IHC is typically required to establish the diagnosis of HAS. The pathological characteristics and expression of various immunohistochemistry staining for HAS are summarized in **Figure 1**. HAC had diffuse expression of AFP, HepPar-1, glypican 3(GPC3), and spalt-like transcription factor 4 (SALL4) with a moderate sensitivity (27). IHC staining for Carcinoembryonic proteins (AFP, SALL4, and GCP3) shows strong diffuse staining of the hepatoid element, suggesting both hepatoid and intestinal mucin phenotype differentiation (33). The intestinal component usually stains for CDX-2 (33, 38). HepPar-1 and Arginase-1 immunostainings are regarded as highly sensitive and specific markers of HCC, while the positive staining of these markers can be detected in some HAC, causing certain difficulties in distinguishing HAS from HCC (37, 39). Among epithelial markers, CK8/18, CK19, and AE1/AE3 are always positive for hepatoid adenocarcinoma; nevertheless, the expression of CK7, CK14, CK20 rarely appears in HAS (37). It has been reported that staining for CEA, CK19, and CK20 is detected more frequently in HAS than in HCC. Furthermore, palate, lung, and nasal epithelium clone protein (PLUNC) is a good marker for distinguishing HAS from HCC because it is often positive in the papillary and tubular adenocarcinoma components of HAS. Anecdotally, PLUNC-positive tumor cells cannot be stained by AFP (40). Though LIN28 is not as sensitive as SALL4, it is a particular marker (98% specificity) for distinguishing classic HAS from HCCs when combining with SALL4. Other IHC stains for HAS, such as Her-2, alpha 1-antitrypsin (AAT), and alpha 1-antichymotrypsin (ACT), have been reported to be promising in making the diagnosis (30, 41).

### Molecular Characteristics

Limited information can be found in the existing literature on the molecular features of HAS. Consisting with the TCGA database, previous reports uncovered that the most frequent genetic mutation in both HAS and GC tumor samples was TP53 (31, 42, 43). RPTOR, CD3EAP, CEBPA, WISP3, and MARK1 other than TP53 were high-frequency gene alternations in HAS (29, 43). It is of note that CTNNB1 and KRAS mutation might be detected in HAC, while subsequent researchers surmised that CTNNB1, KRAS, or BRAF mutations do not exist in most HAC. In addition to gene mutation, HAS is a tumor with a remarkable augment of copy number gains (CNGs). Primarily, the HAS patients with CNGs situated in 20q11.21–13.12 of a chromosome, with a trend of increasing serum concentration of AFP, might be related to more adverse bio-behavior than nonamplified tumors, including lower differentiation, greater nerve and vascular invasion, and more significant liver metastasis and is associated with worse prognosis (29, 42, 43). Moreover, the signaling pathway, including ErbB, PI3K-Akt, HIF-1 and p53 pathway regulating the pluripotency of stem cells, were specifically enriched in the mutated genes. In terms of Epigenetic modifications, GATA4 is not responsible for forming and maintaining the hepatocellular carcinoma-like phenotype (44).

### Serum Tumor Markers

**Figure 2:**
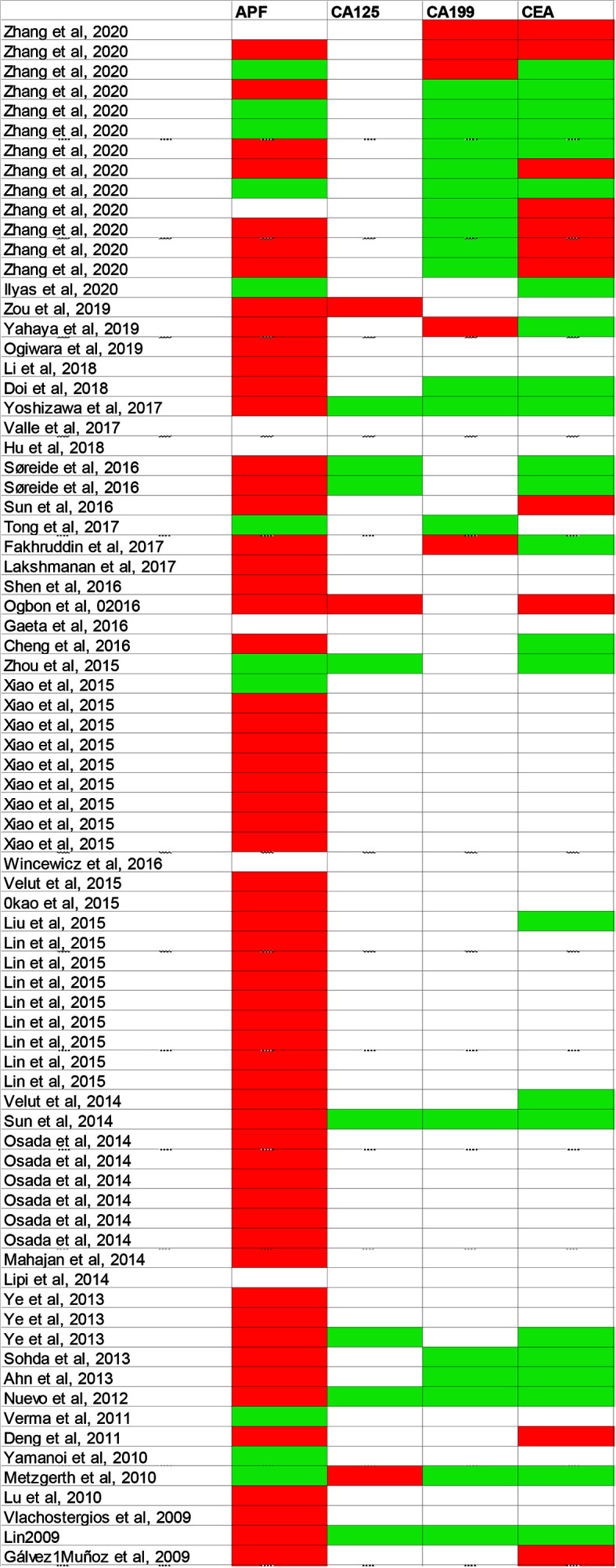
Summarized various of serum tumor markers in published case reports. Diagnostic markers include AFP, CEA, CA19-9 and CA125. White blocks mean this examination has not been mentioned in case reports; green blocks represent negative results; red blocks represent positive results. AFP, Alpha-fetoprotein; CEA, Carcinoembryonic antigen; CA19-9, Carbohydrate antigen 199; CA125, Carbohydrate antigen 125.

The majority of cases reported the elevations in AFP concentration in patients with HAS (**Figure 2**), and the serum AFP concentration was associated with HAC cell component percentage: the higher HAC cell component ratio in a tumor, the more AFP could be secreted by the tumor (22, 42). Although a majority of cases reported the patient had been diagnosed as HAS with the elevation of serum AFP (22), it is of note that there were still patients with HAS whose serum AFP levels were negative despite pathological results that confirmed the presence of Hyaline globule and canalicular structures morphologically (26). Accordingly, HAS's clinicopathological entity was extended, involving adenocarcinomas performing histological patterns of similarity to HCC morphologically regardless of AFP expression/production (36, 39, 45). Other hematological markers, such as the concentration of CA19-9, CA125, CEA, and CA72-4 in the blood, were also elevated in some cases.

## Imaging Diagnosis

For primary sites, the findings of computed tomography (CT), covering the longest and mean short diameter of malignancy, the ratio of lesion attenuation to aorta CT attenuation, the ratio of the number of accrete lymph nodes (LNs) on CT to the number of histologically proven metastatic LNs and the strengthening indexes in arterial phase minus portal venous phase, were significant predictors for distinguishing HAS from other gastric cancer (46–48). For HAC liver metastasis, arterial phase hypo-enhancement was more frequently encountered than HCC. Furthermore, the diffusion-weighted magnetic Resonance Imaging (MRI) was performed for a suspected HAS and clarified the diagnosis of HAS (49). The significance of positron emission tomography (PET)/CT had in diagnosing and staging HAS accurately (50–52).

## Clinical Presentations

HASs were often diagnosed at an advanced disease stage with lymphatic permeation, blood vessel, and regional lymph node metastasis. Among retrospective analysis, 61.5% of HAC patients were in the III or IV stages at the diagnosis time. The relapse rate of early-stage or locally advanced stage patients was 47% (53, 54). The most common sites in which HAC developed include LNs, liver, lungs, peritoneum, and the spleen from existing literature (2, 37). Lacking specific clinical symptoms, the clinical manifestation of HAS is similar to common gastric cancer with many initial symptoms cover epigastric pain (55), abdominal distention (8), backache (55), fatigue (56), reduced appetite, weight loss (57), hematochezia, hematemesis (57) and shortness of breath (58). The most common presentation of HAS is abdominal pain (**Table 1**). Moreover, paraneoplastic hypercholesterolemia has been demonstrated in one case of HAS accompanied by liver metastasis (76).

**Table 1:** Baseline Characteristics.

	Sex/age	Family history	Tumor location	Clinical Manifestation	Lymph nodes	Liver met	TNM	Clinicopathologic stag	Surgery	Treatment except surgery	Survival	Progression	PFS (month)
Zhang et al. (26)	M/68	NO	Antrum	NA	NO	NO	T4aN3aM0	IIIB	YES	5-FU	YES	NO	56
Zhang et al. (26)	M/63	NO	Cardia	NA	YES	NO	T4aN2M0	IIIA	YES	5-FU	NO	YES	28
Zhang et al. (26)	M/58	NO	Body	NA	YES	NO	T2N0M0	IB	YES	5-FU	YES	NO	56
Zhang et al. (26)	M66	NO	Body	NA	NO	NO	T4N0M0	IIB	YES	5-FU	NO	YES	27
Zhang et al. (26)	M59	NO	Antrum	NA	YES	NO	T4N1M0	IIIB	YES	5-FU	NA	NO	NA
Zhang et al. (26)	F/55	NO	Antrum	NA	NO	NO	T4N0M0	IIB	YES	5-FU	YES	YES	56
Zhang et al. (26)	M/60	NO	Antrum	NA	YES	NO	T4N3bM1	IV	YES	5-FU	NO	YES	32
Zhang et al. (26)	F/85	NO	Antrum	NA	NO	NO	T4aN3aM0	IIIB	YES	NO	NO	YES	6
Zhang et al. (26)	M/70	NO	Antrum	NA	YES	NO	T4N3bM0	IIIC	YES	5-FU	YES	YES	23
Zhang et al. (26)	M/74	NO	Antrum	NA	YES	NO	T4bN2M0	IIIB	YES	NO	NO	YES	1
Zhang et al. (26)	M/71	NO	Antrum	NA	YES	NO	T4bN1M0	IIIB	YES	5-FU	NA	NA	NA
Zhang et al. (26)	F/66	NO	Body	NA	YES	NO	T3N1M0	IIB	YES	NO	NA	NA	NA
Zhang et al. (26)	M/64	NO	Cardia	NA	NO	NO	T3N3bM0	IIIC	YES	5-FU	YES	YES	11
Ilyas et al. (59)	M/62	NA		shortness of breath; loss of appetite/weight	YES	NO	YpT3N2R0	NA	NA	L-OHP + Cap RT	NO	YES	12
Zou et al. (8)	M/26	HBV	Peritoneum	abdominal distension	NA	YES	NA	NA	NA	L-OHP+ Cap+ Sorafenib+XELOX+PD-L1	YES	YES	8Circle
Yahaya et al. (5)	M/26	NA	Gastroesophageal junction	loss of appetite/weight epigastric pain	YES	YES	NA	IV	NO	NO	NA	NA	NA
Ogiwara et al. (7)	M/62	NA	Colon	hematemesis/melena diarrhea	NA	YES	T4aN2aM1a	IVA	NA	L-OHP + Cap+ bevacizumab	NO	YES	5
Li et al. (60)	M/60	NA	Colon	hematemesis/melena abdominal distension	YES	NO	T2N1Mx	NA	R2	RT	NA	NA	NA
Yoshizawa et al. (55)	M/61	NA	Antrum	upper abdominal and lower left back pain	YES	YES	T4N2M1	IV	YES	FT/ CDHP/ S-1	YES	YES	2
Valle et al. (1)	M/61	NA	**Lung**	left-sided chest pain	NA	YES	NA	IVB	NO	IMRT	NO	YES	12
Hu et al. (61)	M/63	NO	Gastric	Abdominal distention swelling of his bilateral lower extremities, jaundice, and dark urine, fatigue, melena, loss of weight	NA	NO	NA	IVB	NO	NO	NO	YES	YES
Søreide et al. (56)	M/49	NA	Gastric	fatigue, epigastric discomfort, nausea, anemia	YES	NO	T4bN1M0	NA	YES	NO	NO	YES	3
Søreide et al. (56)	F/81	NA	NA	hematemesis/melena loss of appetite/weight	NA	NO	NA	NA	NO	NO	NO	YES	7
Sun et al. (62)	M/66	NA	Antrum; Body	retrosternal pain.	YES	NO	T3N2M0	IIIB	YES	L-OHP+5-Fu+Ca; TAX+ Cap#	NA	NA	NA
Tong et al. (11)	M/56	NA	NA	hematemesis/melena	NA	NA	T3N1	NA	NO	YES	NO	YES	9
Fakhruddin et al. (63)	F/41	NO	Antrum	abdominal distension epigastric pain	YES	NA	NA	NA	NO	DCX+ Trastuzumab	NO	YES	18
Lakshmanan et al. (64)	M/75	NA	Antrum	fatigue epigastric pain	NO	NO	NA	NA	D2	NO	YES	NO	NA
Shen et al. (65)	M/70	NA	Antrum	muscle weakness; palpitations	NO	YES	NA	NA	YES	L-OHP + Cap#	YES	NA	NA
Ogbonna et al. (6)	M/66	NO	Duodenum	nausea, vomiting, constipation loss of appetite/weight epigastric pain	NA	YES	NA	IV	NO	NO	NO	YES	1
Gaeta et al. (66)	M/72	NA	NA	Fatigue	NA	NO	T3N2M0	IIIB	YES	NA	NA	NA	NA
Cheng et al. (57)	M/83	NA	NA	hematemesis/melena loss of appetite/weight	YES	YES	T3N3M1	IV	NO	NO	NA	NA	NA
Zhou et al. (67)	F/72	NO	Antrum	abdominal distension	YES	NA	NA	NA	YES	L-OHP+ 5-FU+ olinic acid,	YES	NO	NA
Xiao et al. (68)	M/47	NA	Body/	abdominal distension	NA	NO	pT2aN3aM0	IIIA	D2	SOXx6	YES	NO	NA
Xiao et al. (68)	M/63	NA	Antrum/5*3	abdominal distension	NA	NO	pT4aN3bM0	IIIC	D2	FOLFOXx4/#, TS-1	YES	YES	4
Xiao et al. (68)	F/76	NA	Cardia/7*5*3	abdominal distension	NA	NO	pT1bN0M0	IA	D2	NO	YES	NO	NA
Xiao et al. (68)	M/61	NA	Antrum/6.5*4	abdominal distension	NA	NO	pT4aN2M0	IIIB	D2	SOX/#	YES	YES	18
Xiao et al. (68)	M/69	NA	Antrum/3*2.5		NA	NO	pT3N1M0	IIB	D2	Cap+ TAX	YES	YES	11
Xiao et al. (68)	M/57	NA	Antrum/3*4	abdominal distension	NA	NO	pT4aN3M0	IIIC	D2	SOX/#	YES	NO	NA
Xiao et al. (68)	M/67	NA	Cardia/4*3.2	abdominal distension	NA	NO	pT4aN3M0	IIIC	D2	SOX	YES	NO	NA
Xiao et al. (68)	M/58	NA	Antrum/4.5*4	abdominal distension	NA	NO	pT4aN2M0	IIIB	D2	SOX	YES	YES	22
Xiao et al. (68)	M/72	NA	Antrum/4*6	abdominal distension	NA	NO	pT4aN2M0	IIIB	D2	NO	YES	YES	1
Wincewicz et al. (69)	F/73	NA	Gastric/4*6		YES	YES	pT3N3am1	IV	NA	NA	NA	NA	NA
Velut et al. (49)	M/63	NA	**Distal stomach**	abdominal pain	NA	NA	pT2N1M0	NA	YES	FOLFOX	YES	NO	NA
Nakao et al. (70)	M/63	NA	**Body**	positive fecal occult blood	NA	NO	NA	IB	D2	S-1+ CDDP	NA	NA	NA
Liu et al. (34)	M/47	NA	**NA**	upper abdominal ache, nausea, vomiting, melena	YES	NO	NA	NA	YES	Chemotherapy+ radical	YES	NO	NA
Lin et al. (71)	M/64	NA	**Body; Antrum**	Epigastric discomfort	YES	YES	NA	NA	YES	Chemotherapy+ TACE	NO	YES	19
Lin et al. (71)	M/69	NA	**Antrum**	Body weight loss	NA	YES	NA	NA	YES	Chemotherapy	NO	YES	3
Lin et al. (71)	M/78	NA	**Antrum**	Epigastric discomfort	YES	YES	NA	NA	NO	Chemotherapy	NO	YES	5
Lin et al. (71)	M/63	NA	**Cardia**	Epigastric discomfort	YES	YES	NA	NA	NO	Chemotherapy+ TACE	NO	YES	6
Lin et al. (71)	F/70	NA	**Body; Antrum**	Palpable mass	YES	YES	NA	NA	NO	Chemotherapy+ TACE	NO	YES	23
Lin et al. (71)	F/69	NA	**Body; Antrum**	Epigastric discomfort	YES	YES	NA	NA	NO	Chemotherapy	NO	YES	9
Lin et al. (71)	M/60	NA	**Antrum**	Epigastric discomfort	YES	YES	NA	NA	NO	Chemotherapy	NO	YES	3
Lin et al. (71)	M/75	NA	**Body**	Body weight loss	YES	YES	NA	NA	NO	NO	NO	YES	3
Velut et al. (72)	M/63	NA	**NA**	Epigastric pain, weight loss, anemia	YES	NA	T2N1	NA NA	YES	FOLFOX#	YES	NO	NA
Sun et al. (50)	M/73	NA	**NA**	upper abdominal pain	YES	NA	T2N1M0	NA	NA	FOLFOX4	YES	NO	NA
Osada et al. (45)	F/66	NA	**Body/5**	Epigastric pain	NA	YES	NA	NA	NA	NA	NO	YES	13
Osada et al. (45)	M/63	NA	**Body/3.5**	Epigastric pain	NA	NA	NA	NA	NA	NA	YES	NA	NA
Osada et al. (45)	M/61	NA	**Antrum/3.5**	Epigastric pain	NA	NA	NA	NA	NA	NA	YES	NA	NA
Osada et al. (45)	M/78	NA	**Antrum/7**	Epigastric pain	NA	NA	NA	NA	NA	NA	NA	NA	NA
Osada et al. (45)	M/61	NA	**Body/7**	Fatigue, weight loss	NA	YES	NA	NA	NA	NA	YES	NA	NA
Osada et al. (45)	M/75	NA	**Diffuse/3.2**	Fatigue, weight loss	NA	YES	NA	NA	NA	NA	NO	YES	3
Mahajan et al. (73)	M/60	NA	Antrum	pain abdomen	NA	NO	NA	NA	D2	Chemotherapy	YES	NA	NA
Lipi et al. (74)	M/50	NA	NA	Pain abdomen	YES	NA	NA	NA	NA	NA	NA	NA	NA
Ye et al. (75)	F/58	NA	NA	NA	NO	YES	T2N0M1	NA	YES	L-OHP+ Cap, TACE, CT-guided radiofrequency ablation	YES	NO	NA
Ye et al. (75)	M/54	NA	Gastroesophageal junction/4	retrosternal pain	NO	NO	pT2N0M0	IB	YES	L-OHP + 5-FU/#	NO	YES	18
Ye et al. (75)	F/61	NA	NA	epigastric pain, weight loss	NA	NA	NA	NA	NA	L-OHP + S-1	NO	YES	8
Sohda et al. (76)	M/67	NO	Body ; Antrum	NA	NA	YES	NA	NA	NA	NA	NO	YES	2
Ahn et al. (24)	M/68	HBV	Antrum	NA	NA	YES	NA	NA	YES	TS-1/adjuvant Cap+ CDDP/4M, FOLFIRI	YES	NO	NA
Nuevo et al. (77)	F/67	Helicobacter pylori/2y	Antrum/3	fatigue, anorexia, weight loss, anemia	NA	NA	NA	NA	YES	CDDP+ EPI+ Cap/#	NA	YES	12
Verma et al. (78)	M//59	NF-1	Cardia/4	anemia	YES	NO	NA	NA	NA	NA	NA	NA	NA
Deng et al. (79)	M/49	NA	Body/6	NA	YES	NA	pT3N2M1	NA	Subtotal/D4	NA	NA	NA	NA
Yamanoi et al. (80)	M/100	NA	Body	NA	NA	YES	NA	NA	distal	NA	NA	NA	NA
Metzgeroth et al. (41)	M/21	NA	NA	abdominal distension, dyspnea, abdominal pain, weakness, weight loss	NA	NA	NA	NA	NO	TAX+ CBP	NO	YES	6
Lu et al. (81)	M/59	NA	Cardia	melena	YES	YES	NA	NA	total	TACE	NA	YES	6
Vlachostergios et al. (82)	F/85	NA	Antrum/7	epigastric and right upper quadrant abdominal pain, weight loss	NO	YES	NA	NA	NA	NA	NO	YES	4
Lin et al. (83)	F/56	HBV	Body	abdominal dull pain, weight loss	NA	NA	NA	NA	NA	MMC+ 5-FU+ ADM	NO	YES	20
Gálvez-Muñoz et al. (84)	M/75	NA	Cardia; Gastroesophageal junction	abdominal pain, general fatigue, anorexia, sickness	NA	NA	NA	NA	NA	NA	NA	NA	NA

## Treatment

### Surgery

For patients with early-stage HAS, radical surgery is a cornerstone of therapy with curative intent (21, 35). Radical surgery in combination with adjuvant chemotherapy is regarded as the optimal treatment approach (2). Gastric and liver metastasis resection is occasionally performed for palliation in advanced/metastatic HAS patients (85). And it was suggested that salvage surgery following chemotherapy could achieve curative resection of HAS with portal vein tumor thrombus (PVTT) (70).

### Chemotherapy

No standard therapies for HAS were recommended by randomized controlled trials currently. Although the feasibility of neoadjuvant or adjuvant therapy for HAS patients and indications and concrete proposals for auxiliary treatments is illegible (21), adjuvant chemotherapy has been reported as one of the independent factors for a better outcome (35, 68) especially for HAS patients diagnosed with LNs or/and distant organ metastasis (2, 68). It was also reported that FOLFOX might be a potential adjuvant therapy for HAS (72). Cisplatin-based chemotherapy is judged as a standard first-line systemic regimen for metastatic HAS (55). Two advanced HAS patients treated with a first-line chemotherapy regimen of cisplatin and etoposide achieved a complete response (21, 86). The effectiveness of other regimens like oxaliplatin, irinotecan, gemcitabine, and 5-FU, as the first- or second-line treatment, either alone or combined, for advanced HAS situations remains obscure (86).

### Interventional Therapy

Transcatheter arterial chemoembolization (TACE)/hepatic arterial infusion chemotherapy (HAIC), local intra-arterial chemotherapy for liver metastasis of HAS, has a lower frequency of toxicity reactions than systemic chemotherapy because of high concentrations of the drug injected locally (87). Both are also effective for the remission of the liver nodules of mHAS, accompanied with radical surgery or/and systemic chemotherapy.

### Radiotherapy

Radiotherapy (RT) may be an inappropriate therapeutic option for HAS patients due to limited efficacy data. A scarce event reported that one patient with HAC of lung metastasizing to tonsils obtained an extraordinary symptomatic remission after the therapy of intensity-modulated radiation therapy (IMRT) (1). The palliative fractionation of RT was delivered to patients with PS (≧2) purely for symptom control, developing an unusual radiological adverse reaction to RT (59).

### Anti-Angiogenesis Drugs

The introduction of anti-angiogenesis drugs has expanded treatment options of HAS. A case demonstrated that a HAS patient's resistance to chemotherapy had an evident clinical response to ramucirumab (RAM) monotherapy (87). The AFP concentration might be a potential marker to predict the response to ramucirumab and other anti-angiogenic drugs in gastric cancer. Besides, the positive Her-2 test rate of HAS patients was around 25%. Combined with chemotherapy, such as capecitabine and cisplatin, Trastuzumab could improve HER2-positive advanced HAS patients' overall survival compared with those who received chemotherapy alone (63, 87–90). Sorafenib, a molecularly targeted drug via the unclear mechanism of its direct pro-apoptotic effects or anti-angiogenic properties, has been administrated in some HAC patients. But it was suspended attributable to early adverse reactions (21). No convincing evidence about the sensitivity of HAS to Sorafenib was reported. In addition, HAC of the ovary and peritoneum were insensitive to Sorafenib (8).

### Immunotherapy

Immune checkpoint antibodies have been approved to be administrated in multiple solid tumors, incorporating carcinomas of lungs, liver, esophagus, kidney, and stomach. Currently, immunotherapy applied to HAS is rare to report. Only one case showed that one HAS patient managed with PD-L1 inhibitor represented a low curative effect, which might be related to its low expression of PD-L1. Further experimental verification is expected to be reached in future clinical trials (8).

## Prognostic Factors

The prognosis of HAS is poor. HAS patients had notably lower survival rates and disease-free survival (DFS) compared to those with other types. It is revealed that the 5-year DFS of HAS patients was only 20.7% (2, 33, 91). It was concluded that pTNM stage, portal vein thrombosis, vascular invasion, and adjuvant treatments were independent risk factors for DFS and pTNM stage, entirely surgical resection, and adjuvant therapy were independent risk factors for disease-specific survival (DSS) (2). However, some case reports argued that survival was not associated with sex, location, type, the serum AFP level, the degree of differentiation, or the type of therapy received. Although the relationship between neuroendocrine differentiation and the prognosis of HAS remained vague, it was inclined to an unfavorable factor to give rise to low differentiation and prognosis (92).

Morphologically, clear cell histology, more than a threshold of 10% about the ratio of clear cells, harmed prognosis in patients within HAS (33, 38). No evidential relations were deemed between immunohistochemical staining and prognosis in HAC. Among epithelial markers, including CEA, CK7 and CK20 were crucial for survival assessment by immunohistochemistry stains (8). Patients with CEA, CK20, and CK7 staining positive lived a shorter life. Furthermore, the combination of PLUNC, SALL4, and Hep-Par-1 might be a way of a tried prognostic factor in HAS (40).

Also, the patients with higher AFP expression had a significantly more inferior OS (58). AFP was assumed to be adverse to tumor suppression due to inhibiting lymphocyte transformation (27). However, The AFP-positive cases had shown better outcomes than the AFP-negative instances in a series of HAC with enteroblastic differentiation(GAEDs) (43). Meanwhile, It was observed the expression of β-catenin has a significant correlation with survival time (27).

## Future Perspectives

Although the standard surgical and systemic chemotherapies have been proved to improve the prognosis of HAS, it still shows a poor clinical outcome. Cisplatin-based chemotherapy regimens are regarded as the first-line treatments for metastatic HAS, while the second-line systemic approaches for optimal management remain unclear. Further researches should be directed at exploring the radiobiological sensibility and radiational therapeutic effects in these patients (59). A significant step toward applying anti-angiogenesis drugs covering RAM combining with chemotherapy, the overall survival of advanced HAS patients has been significantly increased. Of note, the development of molecularly targeted treatments related to Sorafenib should be validated. Immunotherapy as a possible therapeutic means is to be further explored in patients with HAS.

## Conclusion

HAS is a scare subtype of gastric cancer. It is often diagnosed with lymph node metastasis and distant organ metastasis and has a poor prognosis, which poses a significant challenge to clinicians' diagnosis and treatment. Several immunohistochemical markers covering AFP, CEA, CK8/18, CK19, glypican 3, SALL4, CDX-2, and HepPar-1 can be performed to assist in pathological confirmation. The level of AFP serum is propitious to the early detection of HAS. The available radical surgery, chemotherapy, radiotherapy, and interventional therapy in HAS patients have achieved a better outcome. The introduction of anti-angiogenesis drugs has expanded the therapeutic boxes of HAS. The prognostic risk factors of HAS are related to infiltrating depth, portal vein thrombosis, vascular invasion, distant metastasis, pTNM stage, serum AFP levels, therapeutic regimen, and immunohistochemical staining. Immunotherapy and radiotherapy need to be further validated in HAS.

## Author Contributions

RX collected data, reviewed the literature, and wrote the manuscript. YZ collected data and wrote and revised the manuscript. YW collected data and rechecked the manuscript. JY assisted in drawing. XM designed and revised the manuscript. All authors contributed to the article and approved the submitted version.

## Conflict of Interest

The authors declare that the research was conducted in the absence of any commercial or financial relationships that could be construed as a potential conflict of interest.
